# Functional outcomes of operative fixation of clavicle fractures in patients with floating shoulder girdle injuries

**DOI:** 10.1007/s10195-015-0349-8

**Published:** 2015-05-05

**Authors:** Alex K. Gilde, Martin F. Hoffmann, Debra L. Sietsema, Clifford B. Jones

**Affiliations:** Grand Rapids Medical Education Partners, Orthopaedic Surgery Residency, Grand Rapids, MI 49503 USA; BG-University Hospital Bergmannsheil, Bochum, Germany; Michigan State University College of Human Medicine, 15 Michigan St NE, Grand Rapids, MI 49503 USA; Orthopaedic Associates of Michigan, 230 Michigan St NE, Suite 300, Grand Rapids, MI 49503 USA

**Keywords:** Clavicle, Floating shoulder, Scapula, Osteosynthesis, Outcome

## Abstract

**Background:**

Double disruptions of the superior suspensory shoulder complex, commonly referred to as ‘floating shoulder’ injuries, are ipsilateral midshaft clavicular and scapular neck/body fractures with a loss of bony attachment of the glenoid. The treatment of ‘floating shoulder’ injuries has been debated controversially for many years. The purpose of this study was to demonstrate the clinical and functional outcomes of patients with ‘floating shoulder’ injuries who underwent operative fixation of the clavicle fracture only.

**Materials and methods:**

Between 2002 and 2010, 32 consecutive floating shoulder injuries were identified in skeletally mature patients at a level I trauma center and followed in a single private practice. Thirteen patients met the inclusion and exclusion criteria for this retrospective study with a minimum 12-month follow-up. Clavicle and scapular fractures were identified by Current Procedural Technology codes and classified based on Orthopaedic Trauma Association/Arbeitsgemeinschaft für Osteosynthesefragen criteria. ‘Floating shoulder’ injuries were surgically managed with only clavicular reduction and fixation utilizing modern plating techniques. Nonunion, malunion, implant removal, range of motion, need for secondary surgery, pain according to the visual analog scale (VAS), and return to work were measured.

**Results:**

All injuries were the result of high-energy mechanisms. Fracture union of the clavicle was seen after initial surgical fixation in the majority of patients (12; 92.3 %). Final pain was reported as minimal (11 cases; 1–3 VAS), moderate (1 case; 4–6 VAS), and high (1 case; 7–10 VAS) at last follow-up. Excellent range of motion (180° forward flexion and abduction) was observed in the majority of patients (8; 61.5 %). The Herscovici score was 12.9 (range 10–15) at 3 months. Unplanned surgeries included two clavicular implant removals and one nonunion revision. None of the patients required reconstruction for scapula malunion after nonoperative management. Twelve patients returned to previous work without restrictions.

**Conclusions:**

‘Floating shoulder’ injuries with only clavicular fixation return to function despite persistent scapular deformity and some residual pain.

*Level of evidence* Level IV.

## Introduction

Double disruptions of the superior suspensory shoulder complex (SSSC) resulting in ipsilateral midshaft clavicular and scapular body/neck fractures, are commonly referred to as a ‘floating shoulder’ injury, and result in a loss of bony attachment of the glenoid [1, 2]. Floating shoulder injuries are the result of high-energy mechanisms [3–5] with an incidence of approximately 0.10 % of trauma patients [6]. Although much is known about these fractures when they occur in isolation, evidence is lacking in regard to treatment as concomitant fractures are associated with poor cosmesis, reduced strength, and dyskinesia of the shoulder girdle. Ganz and Noesberger [7] originally suggested that the weight of the arm and the muscles at the humerus would cause caudal and anteromedial displacement of the glenoid. Ada and Miller [3] found high numbers of rotator cuff dysfunction in patients with displaced clavicular and scapular fractures, as the normal lever arm of the rotator cuff is lost with glenoid displacement.

The treatment of ‘floating shoulder’ injuries has been debated over many years. Several studies recommend that conservative treatment results in acceptable patient outcomes, especially when fractures are minimally displaced [8–13]. Other studies have reported good to excellent outcomes with only clavicular fixation [6, 14–17]. In floating shoulder injuries with significant displacement, some studies have recommended fixation of both clavicular and scapular fractures [11, 13, 18–20]. Despite cited surgical indications for isolated extra-articular and intra-articular scapular fractures [3, 21–25], validated indications for ‘floating shoulder’ surgical management remain unclear. The purpose of this study was to describe the clinical and functional outcomes of patients with displaced and unstable ‘floating shoulder’ injury following fixation of only the clavicular fracture.

## Materials and methods

This Institutional Review Board-approved retrospective exploratory study reviewed operatively treated midshaft clavicular fractures with associated ipsilateral non-operatively treated scapular fractures. The patients were recruited from a private practice office associated with a level I teaching trauma center. Consecutive patients were identified using Current Procedural Technology coding for operatively fixed clavicle fractures (23515) and a scapular injury database from March 1, 2002 to October 1, 2010.

Operative criteria for clavicular fixation included significant clavicular shortening (>20 mm on either anterior-posterior [AP], cephalad, or caudal radiographs), associated neurological injury, associated unstable scapular injury (glenoid neck, acromion, coracoid, or intra-articular glenoid fractures), double suspensory shoulder instability, open clavicular fractures, published criteria for displacement, impending skin compromise, or polytrauma [26–30]. Inclusion criteria for this study were skeletally mature (age ≥18 years), ipsilateral middle third clavicular fracture and scapular fracture meeting the definition of a ‘floating shoulder’, clavicle fixation utilizing modern plating techniques [27, 31–33], and a minimum 12-month follow-up. A total of 32 patients were identified during this time period. Nineteen patients were excluded due to initial non-operative treatment of the clavicle fracture with subsequent nonunion (1), incarceration (1), insufficient records or imaging (7), and operative treatment of both clavicle and scapular fractures (10). When initially planning surgical management of these patients, the indications for fixation of the scapular fracture were partly based on the preferences of the senior surgeons as well as the patient’s clinical condition. All patients in this study, with the exception of two, did not meet currently published indications for fixation of the scapular fracture in ‘floating shoulder’ injuries [11]. Thirteen ‘floating shoulder’ injuries in 13 patients formed the basis of this study.

**Fig. 1:**
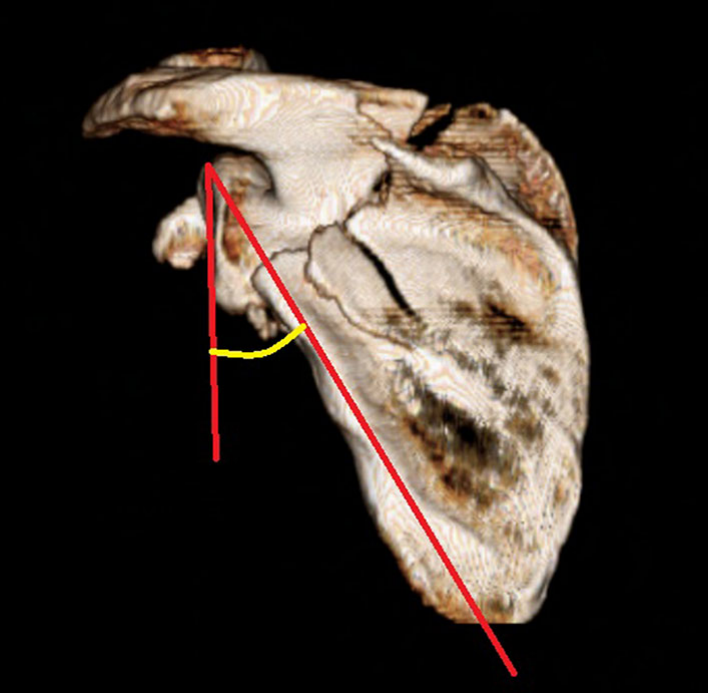
The glenopolar angle as measured on 3D CT reconstruction. The apex created by *two lines* extending from the superior glenoid pole to the mid-point of the inferior angle and inferior glenoid pole determine the glenopolar angle

**Fig. 2:**
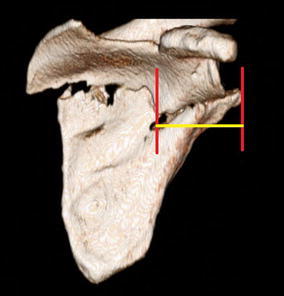
Medialization/lateralization displacement as measured on 3D CT reconstruction. It is determined by the distance between the *vertical planes* drawn at the lateral-most edge of both scapular fragments

All patients were treated and followed by four fellowship-trained orthopedic trauma surgeons utilizing similar philosophies and techniques. At the time of injury, all patients had computed tomography (CT) scans with three-dimensional (3D) reconstruction of the scapular fracture to assess deformity which included the glenopolar angle [34] (Fig. 1) and medialization/lateralization [35] (Fig. 2) of the scapular fragments [GE LightSpeed VCT 64-slice CT scanner; GE Healthcare, Waukesha, WI, USA (1.25-mm slice thickness); 3D reconstruction with TeraRecon Aquarius iNtuition v.4.4.5.49; TeraRecon, Inc, Foster City, CA, USA].

At 1–2 weeks postoperatively, physical therapy-directed passive range of motion (ROM) was instituted in all patients. At 6 weeks postoperatively, physical therapy-directed active ROM and strengthening was started. Patients were evaluated and imaged at regular intervals of 2, 6, and 12 weeks, and ongoing according to clinical necessity including, but not limited to pain, plate irritation, plate prominence, poor ROM, or not achieving complete clinical healing. Cephalad and caudal views of the clavicle were obtained at each interval to determine healing and alignment [36]. Grashey (true shoulder AP), axillary, and scapular Y views of the shoulder were obtained at each interval to determine scapular healing and morphology. Distances and angles were measured using digital software with a picture archiving and communication system or by manual techniques and protractors. Both clavicle and scapular fracture patterns were classified according to Orthopedic Trauma Association/Arbeitsgemeinschaft für Osteosynthesefragen (OTA/AO) criteria [37].

Pain according to the visual analog scale (VAS) [38] and ROM using basic clinical measurements were recorded. Outcomes were further measured using the Herscovici scoring system which assigns a numerical value (1–4) for pain, lifestyle, ROM, and muscle strength with a value of 16 being the best possible outcome [6]. Return to previous work was assessed. Follow-up was for a minimum of 12 months with radiographic union and return to previous activities and/or employment being established.

Standard statistical analyses were employed. Descriptive statistics, including means, range, standard deviation, and percentages were calculated using SPSS^®^ 18.0 (IBM, Armonk, NY, USA).

## Results

**Fig. 3:**
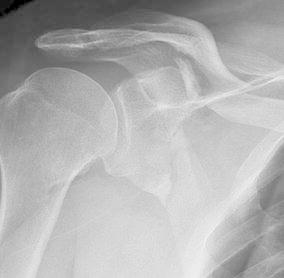
This patient sustained 15-B2 clavicular and 14-A3.1 scapular fractures in a motorcycle accident

The mean follow-up was 16 months (12–45 months). The mean age at time of injury was 46 years (18–60 years) and 10 patients were male. High-energy mechanisms were the cause of all patient injuries including motorcycle accidents (11; 84.6 %) and all-terrain vehicle accidents (2; 15.4 %). None of the fractures were classified as open. Clavicular fracture classification was recorded as type 15-B1 (5; 38.4 %), type 15-B2 (7; 53.9 %), and type 15-B3 (1; 7.7 %). Scapular fracture classification was recorded as type 14-A3.1 (7; 53.9 %), type 14-A3.2 (5; 38.4 %), and type 14-C1.1 (1; 7.7 %) (Fig. 3). All clavicle fractures were unstable with shortening averaging 14 mm (6–30 mm) and translation averaging 10 mm (2–24 mm). Associated injuries were found in 12 of the 13 patients (92.3 %), which included rib fractures (11; 84.6 %), ipsilateral extremity fractures (6; 46 %), pneumothorax (5; 38.4 %), intracranial hemorrhage (2; 15.4 %), and abdominal hemorrhage/laceration (2; 15.4 %). Injury and fracture data are displayed in Table 1.

**Table 1:** Patient demographic and injury data

Case	Age	Gender	Clavicle fracture classification	Scapula fracture classification	Clavicle translation; shortening (mm) +/−	Glenopolar angle (°)	Scapular medialization/lateralization (mm)	Associated injuries
1	45	F	15-B1	14-A3.2	8; +17	41	23	1, 3, 4
2	54	M	15-B2	14-A3.1	8; −16	34	0	2, 3
3	45	M	15-B1	14-A3.2	0; +21	34	15	1
4	54	M	15-B2	14-A3.1	2; −8	32	4	1
5	48	M	15-B2	14-C1.1	16; +19	49	0	1
6	41	F	15-B1	14-A3.1	8; −6	44	9	1
7	60	M	15-B2	14-A3.1	17; −12	34	24	1, 2, 3, 4, 5
8	18	M	15-B2	14-A3.2	24; −9	48	6	
9	45	M	15-B3	14-A3.1	4; −8	40	10	1, 2, 3
10	44	M	15-B2	14-A3.1	7; −7	38	42	1, 5
11	40	F	15-B1	14-A3.1	21; −10	34	17	1, 2
12	51	M	15-B1	14-A3.2	10; −15	28	31	1, 2, 3
13	52	M	15-B2	14-A3.2	7; +30	40	28	1, 2

*1* rib fracture(s), *2* ipsilateral extremity fractures, *3* pneumothorax, *4* intracranial hemorrhage, *5* abdominal hemorrhage/laceration

Eleven of 13 (85 %) patients reported minimal pain (VAS 1–3) upon final examination. One patient (1; 7.7 %) reported moderate levels of pain (VAS 4–6), which was attributed to overlying skin irritation at the surgical site. One patient (1; 7.7 %) reported high levels of pain (VAS 7–10) which was associated with the development of a nonunion. All patients eventually returned to work, 12 of whom had no restrictions. One patient (case 13) returned to function with restrictions secondary to pain despite complete nonunion resolution and full symmetrical strength and ROM.

Eight of 13 patients (62 %) had full symmetrical ROM (180° of forward flexion and abduction) at last follow-up. Three patients without complete restoration of ROM showed adequate ROM and function necessary to perform activities of daily living of the shoulder joint (flexion >121°, abduction >128°) [39]. Two patients exhibited suboptimal ROM at last follow-up. One of these patients (case 7) had a traumatic brain injury that impeded formalized therapy and therapy compliance. The mean Herscovici score for all patients was 12.9 at 3-month follow-up (range 10–15). Patient outcome data is categorized in Table 2.

**Table 2:** Patient outcomes

Case number	Final follow-up					3 months
	Forward flexion	Abduction	Pain	Subsequent surgery	Return to work	Herscovici score^a^
1	180	180	Minimal	None	Full return	15
2	180	180	Minimal	None	Full return	10
3	180	180	Minimal	None	Full return	13
4	180	180	Minimal	None	Full return	14
5	150	140	Minimal	None	Full return	15
6	150	150	Minimal	None	Full return	13
7	180	120	Minimal	None	Full return	11
8	160	160	Minimal	None	Full return	15
9	180	180	Minimal	Implant removal	Full return	14
10	140	110	Minimal	None	Full return	11
11	180	180	Moderate	Implant removal	Full return	14
12	180	180	Minimal	None	Full return	13
13	180	180	High	Nonunion revision	Restrictions	10

Minimal, moderate, and high pain levels correspond to VAS of 1–3, 4–6, and 7–10, respectively

^a^Mean Herscovici for all patients at 3 months was 12.9

Twelve of 13 (92 %) clavicular fractures initially healed. One infected nonunion successfully healed after debridement, antibiotics, and revision plating. All of the scapular fractures healed with radiographic evidence of malunion without further displacement. None of the scapular fractures required reconstructive surgery to realign the scapular malunion after initial conservative management.

## Discussion

Stable, minimally displaced isolated clavicular and scapular fractures heal quickly and predictably with conservative nonoperative treatment [13, 40–42]. These injuries, however, are different from the unstable, displaced ‘floating shoulder’ injuries [1, 2]. ‘Floating shoulder’ injuries are rare with complex fracture patterns. This type of double SSSC injury is usually the result of high-energy trauma and often has associated ipsilateral shoulder and chest trauma.

Previous studies have described clinical outcomes following clavicular fixation of these injuries with varied results (Table 3). Herscovici et al. [6] reported on seven patients who had excellent outcomes with a Herscovici score of 13–16 after surgical fixation of only the clavicle. Two conservatively treated patients had persistent shoulder ‘drooping’, but could not undergo operative treatment due to severe injuries. An alternative randomized study of 25 patients by Yadav et al. [17] reported a significantly greater mean Herscovici score at 3 and 24 months in patients treated with clavicular fixation only compared to conservative management (13.9 vs 10.4 and 14.9 vs 13.0, respectively). Labler et al. [11] reported on 17 patients treated either conservatively, with clavicular fixation only, or with combined clavicular and scapular fixation. In their operative group, five patients showed good to excellent results (Constant–Murley scores 93–100) and four patients showed bad to fair results (Constant–Murley scores 0–86). The high Constant–Murley scores seen in the nonoperative group correlated with minimally displaced clavicular and scapular fractures suggestive of stable patterns. Van Noort et al. [12] reported that only two of seven patients had a corresponding indirect scapular reduction with only clavicular fixation, and persistent caudal displacement of the glenoid was observed in the other five patients. Fourteen of 28 conservatively treated patients showed persistent ‘drooping’ of the shoulder. Hashiguchi and Ito [14] found fracture union of all five clavicular and scapular fractures treated with only clavicular plating. Correspondingly high UCLA shoulder scores were noted. Rikli et al. [15] reported healing of 11 clavicular fractures after plating. Nine of their patients were completely pain free at last follow-up. Four patients changed jobs; however, three of these changes were secondary to concomitant injuries. Oh et al. [18] found improved mean Rowe scores in clavicular fractures treated operatively versus conservative management. Low and Lam [16] reported one good (Rowe score 70–84) and three excellent outcomes (Rowe score 85–100) after only clavicular fixation.

**Table 3:**
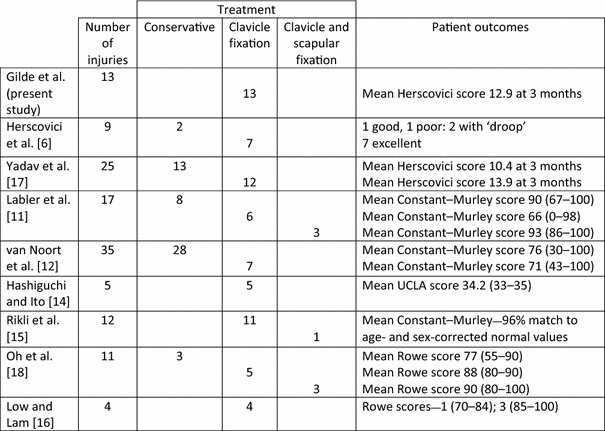
Published results involving only clavicle fixation of floating shoulder injuries

This study also demonstrates that ‘good’ to ‘excellent’ outcomes can be observed with only clavicle fixation in patients with floating shoulder injuries. At 3 months after fixation, we observed a mean Herscovici score of 12.9 consistent with near excellent outcomes. Four patients had good outcomes (9–12) and 9 experienced excellent outcomes (13–16) despite the severity of associated injuries and varying levels of shoulder instability. This does not correspond to the outcomes published by Yadav et al.; however, patients with associated neurovascular injuries or rib fractures requiring intervention were included in this study. We agree with previous studies and the recommendations set forth by Labler et al. that the majority of patients with floating shoulder injuries resulting in minimally displaced scapular fractures that are treated with clavicular plating may return to function despite varying levels of pain and scapular deformity [11]. On the basis of our patient outcomes, we recommend only clavicular fracture fixation for minimally displaced ‘floating shoulder’ injuries.

A weakness of this study is the retrospective design and absence of a standardized functional outcome tool such as the UCLA shoulder score, ASES shoulder scoring scale, Constant–Murley score, and disabilities of arm, shoulder, and hand (DASH) systems. No validated surgical indications for scapular fixation exist in the literature. Without validated surgical indication, surgeon and/or patient preference for nonoperative versus operative management does not exist; therefore, potential patient selection and treatment intervention bias may have occurred. All patterns were complex shoulder girdle injuries with varied stability. The strengths of this study include isolated clavicular fixation of a relatively large number of ‘floating shoulder’ injuries utilizing modern plating techniques. Patients were followed until complete fracture healing and operative site healing became stable.

Further research efforts are needed to reliably quantify scapular deformities objectively in order to determine surgical indications and the effectiveness of postoperative reduction of the glenoid following clavicular fixation. Three questions still persist regarding the ‘floating shoulder’. First, does clavicular fixation actually restore the scapula fracture component to its pre-injury anatomy [43, 44]? Secondly, is residual pain related to the combined shoulder girdle injury, clavicular fixation, and/or the scapular malunion? Lastly, would scapular fracture fixation decrease required formal physical therapy, allow patients to return to work earlier, or improve their overhead strength and endurance.

In conclusion, isolated plate fixation of the clavicular fracture in ‘floating shoulder’ injuries results in high rates of both clavicular and scapular fracture healing with good to excellent outcomes. Despite varying persistent shoulder girdle pain and scapular malunion, the majority of patients returned to previous work.
